# Long noncoding RNA Crnde attenuates cardiac fibrosis via Smad3‐Crnde negative feedback in diabetic cardiomyopathy

**DOI:** 10.1111/febs.14780

**Published:** 2019-03-01

**Authors:** Dezhi Zheng, Yong Zhang, Yonghe Hu, Jing Guan, Lianbin Xu, Wenjing Xiao, Qinyue Zhong, Chao Ren, Jinfeng Lu, Jiali Liang, Jun Hou

**Affiliations:** ^1^ Department of Cardiovascular Surgery The 960th Hospital of the PLA Joint Logistic Support Force Jinan China; ^2^ Department of Pharmacy The General Hospital of Western Theater Command Chengdu China; ^3^ Department of Radiology The General Hospital of Western Theater Command Chengdu China

**Keywords:** cardiac fibrosis, Crnde, diabetic cardiomyopathy, long noncoding RNA

## Abstract

Diabetic cardiomyopathy (DCM)—ventricular dysfunction in the absence of underlying heart disease—is a common complication of diabetes and a leading cause of mortality associated with the disease. In DCM, cardiac fibrosis is the main cause of heart failure. Although it is well‐established that the transforming growth factor‐beta signaling pathway plays a part in inducing cardiac fibrosis in DCM, details of the molecular mechanism involved remain elusive. Therefore, it is crucial to study the gene reg;ulation of key signaling effectors in DCM‐associated cardiac fibrosis. A recently emerged hotspot in the field of gene regulation is the role of long noncoding RNAs (lncRNAs). Recent evidence indicates that lncRNAs play a critical role in cardiac fibrosis; however, in DCM, the function of these regulatory RNAs have not been studied in depth. In this study, we identified a conserved cardiac‐specific lncRNA named colorectal neoplasia differentially expressed (Crnde). By analyzing 376 human heart tissues, it was found that Crnde expression is negatively correlated with that of cardiac fibrosis marker genes. Moreover, Crnde expression was shown to be enriched in cardiac fibroblasts (CFs). Overexpression of Crnde attenuated cardiac fibrosis and enhanced cardiac function in mice with DCM. Further, *in vitro* experiments showed that Crnde negatively regulates the myofibroblast differentiation of CFs. The expression of Crnde was activated by SMAD family member 3 (Smad3), shedding light on the underlying molecular mechanism. Interestingly, Crnde also inhibited the transcriptional activation of Smad3 on target genes, thereby inhibiting the expression of myofibroblastic marker genes in CFs. Overall, our data provide valuable insights into the development of potential anti‐cardiac fibrosis strategies centered on lncRNAs, for the treatment of DCM.

## Graphical abstract

Diabetic cardiomyopathy (DCM) is a common and serious complication of diabetes. Long non‐coding RNAs have previously been linked to cardiac fibrosis; however, the role they play in DCM‐associated cardiac fibrosis remains unclear. In this study, Jun Hou and colleagues identified a cardiac‐specific lncRNA, Crnde, whose expression negatively correlated with cardiac fibrosis markers. Overexpression of Crnde attenuated cardiac fibrosis and enhanced cardiac function in DCM mice. *In vitro*, Crnde negatively regulated the myofibroblast differentiation of cardiac fibroblasts. The team shed light on the molecular mechanism – involving TGF‐β/Smad3 signaling – providing an interesting new avenue for development of anti‐fibrosis strategies for DCM.

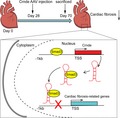

**Abbreviations**
AAV: Adeno‐associated virus
Acta2 (α‐SMA): actin‐alpha 2 (α‐smooth muscle), aorta
CFs: cardiac fibroblasts
CMs: cardiomyocytes
Col1a1: collagen type I alpha 1
Col3a1: collagen type III alpha 1
Crnde: colorectal neoplasia differentially expressed
DCM: diabetic cardiomyopathy
HF: heart failure
MIAT: myocardial infarction‐associated transcripts
RIP: RNA‐binding protein immunoprecipitation
SBE: Smad‐binding element
Smad2/3/4: SMAD family member 2/3/4
TGF‐β: transforming growth factor‐beta

## Introduction

Diabetes is one of the major public health diseases 1. Myocardial disease is the leading cause of morbidity and mortality worldwide in diabetic patients 2. Diabetic cardiomyopathy (DCM), a diabetic complication, causes heart failure (HF). The characteristic feature of DCM is a distinct primary pathology that leads to HF in the diabetic patient. Also, lots of epidemiological evidence have shown an association between HF and DCM 3.

Recent studies have shown that cardiac fibrosis caused by diabetes is a major characteristics of DCM 4. In DCM, excessive production of extracellular matrix (ECM) protein causes an increase in myocardial stiffness, and ultimately leading to HF. Cardiac fibrosis causes pathological changes, leading to ventricular dilatation, cardiomyocyte (CM) hypertrophy, and apoptosis eventually congestive HF 5, 6. Activation of cardiac fibroblasts (CFs) and differentiation into myofibroblasts trigger the pathological process of the DCM. CFs in connective tissue are converted to its activated form, commonly referring to as myofibroblasts 7, 8, 9. Activated myofibroblasts display raised protein synthesis, including collagens and alpha‐SMA (a marker of contractile protein and profibrotic CFs activation) 10.

Because fibrosis plays vital importance in the pathology of DCM, researchers have centered on the molecular mechanisms of myofibrosis in DCM. Studies have indicated enhanced expression level of transforming growth factor‐beta (TGF‐β) in animal experiments of type 1 and type 2 diabetes model 3. TGF‐β is known to mediate tissue inflammation and damage accompanied by tissue fibrosis, and it also plays an essential role in activating CFs. TGF‐β causes fibroblast activation, proliferation, and differentiation into myofibroblasts which secreting ECM proteins 11, 12. A typical pathway for TGF‐β1 signaling involves phosphorylation of SMAD family member (Smad) 2/3, which subsequently binds to Smad4 and causes nuclear translocation. This complex then performs as a specific transcription factor that induces activation of many profibrotic genes 13, 14, 15, 16.

Currently, there is no effective treatment for myocardial fibrosis, partly because the understanding of the mechanism of fibrogenesis is incomplete. Therefore, genetic regulation of cardiac fibrosis could better an understanding of the novel anti‐fibrotic approaches for DCM. Recently, long noncoding RNA (lncRNA) is a hotspot in gene regulation research. lncRNA is the mRNA‐like transcript that is 200–100 kb in length and lacks protein‐encoding activity but is involved in many basic biological developments and pathophysiological incidents 17, 18, 19. Recent studies have demonstated that the expression of different ncRNAs, including lncRNA, are closely linked to the development, progression, and treatment of myocardial fibrosis 20, 21, 22, 23. However, few studies have reported the lncRNA that plays a fundamental role directly related to the strong TGF‐β/Smad3 signal of cardiac fibrosis in DCM.

In the present study, we scanned through a public database and found a CFs‐enriched lncRNA, colorectal neoplasia differentially expressed (Crnde), which negatively associated with the fibrotic marker genes actin‐alpha 2 (α‐smooth muscle), aorta (Acta2 (α‐SMA)). Subsequent experiments demonstrated that lncRNA Crnde negatively regulated CFs activation and myofibroblast differentiation marker gene expression *in vitro*. Meanwhile, overexpression of Crnde by Adeno‐associated virus (AAV) *in vivo* attenuated myocardial fibrosis and enhanced cardiac function in DCM mice. Moreover, this study also conducted an in‐depth investigation of the molecular mechanism of Crnde's anti‐fibrosis function in DCM.

## Results

### Crnde (CRNDE) was a cardiac‐specific lncRNA, which was enriched in CFs

**Figure 1:**
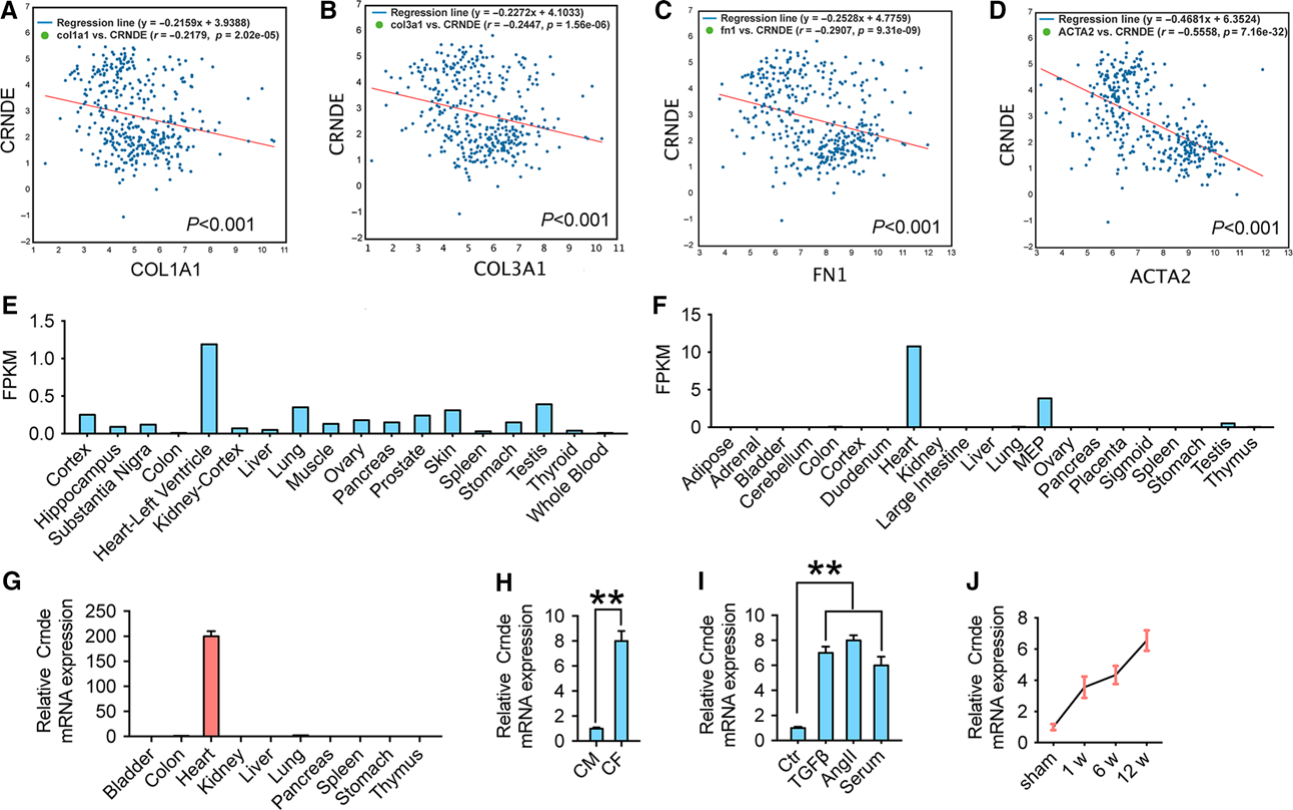
CRNDE is a CFs‐specific lncRNA. There were negative correlations between CRNDE and COL1A1 (A), COL3A1 (B), FN1 (C), and ACTA2 (D) expression in 376 heart tissues in ChIPbase V2 database. (E) Relative expression levels of CRNDE in different tissues of humans. (F) Relative expression levels of Crnde in different tissues of mice. (G) RT‐qPCR detects the relative expression of Crnde in different tissues of mice. (H) RT‐qPCR detection of PFL expression in CMs and CFs. (I) Relative expression levels of Crnde in CFs after treatment with TGF‐β, Ang II, or 20% serum for 24 h. (J) Relative expression of Crnde after 7, 14, and 21 days after the establishment of the MI model. All values are expressed as mean ± SD. ** *P* < 0.01.

The lncRNA Crnde (CRNDE) was found negatively correlated with the myocardial fibrosis marker gene collagen type I alpha 1 (COL1A1) in 376 samples of human heart mixed tissue using the chipbase v2 (http://rna.sysu.edu.cn/chipbase/) website 24 (Fig. 1A–D). Therefore, we suspected that lncRNA CRNDE might have an inhibitory effect on cardiac fibrosis. Then, we used the locexpress (http://loc-express.cbi.pku.edu.cn/submit/new) database 25 to analyze the expression of lncRNA CRNDE in different tissues of humans and mice and found that Crnde (CRNDE) was specifically expressed in cardiac tissues of both humans and mice (Fig. 1E,F). To further validate the specificity of Crnde expression in mouse hearts, we used real‐time quantitative PCR (RT‐qPCR) to measure the expression of Crnde in different tissues of the mouse. The results were similar to human tissue in the database, and Crnde was explicitly expressed in mouse heart tissue, which was significantly different from other tissues (Fig. 1G). Further, the results of qPCR also showed that Crnde was elevated considerably in CFs compared to CMs (Fig. 1H). Interestingly, Crnde in CFs significantly increased after treatment with TGF‐β1 (10 ng·mL^−1^), angiotensin II (Ang II, 100 nm), or 20% serum for 24 h expression (Fig. 1I). To investigate the role of Crnde in cardiac fibrosis, we subsequently used mouse DCM model to induce cardiac fibrosis. Consistent with *in vitro* experiments, Crnde increased significantly in DCM mice in a time‐dependent manner (Fig. 1J).

### Crnde attenuated cardiac fibrosis and enhanced heart function in DCM mice

**Figure 2:**
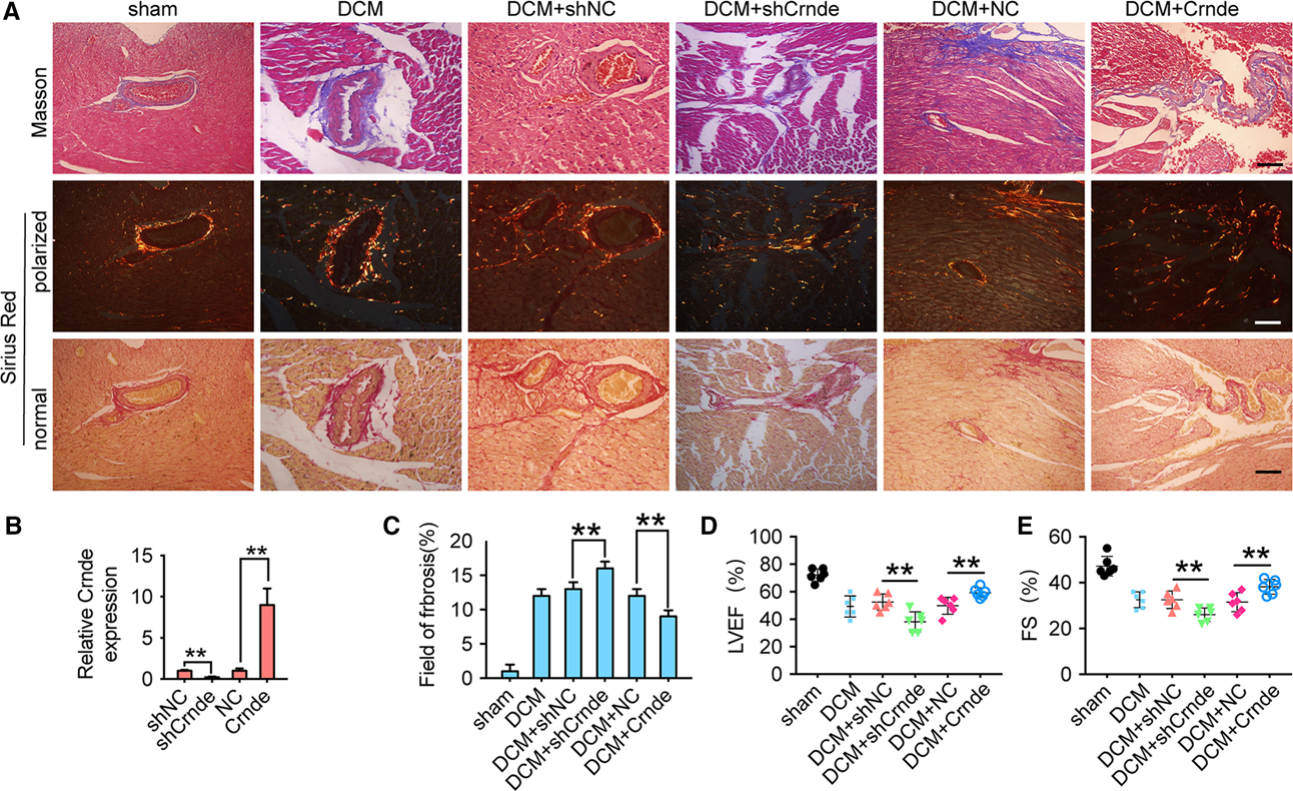
Crnde regulates DCM‐induced myocardial fibrosis. (A) qRT‐PCR analysis showed that adenovirus was able to knock down and overexpress Crnde in cardiac tissue. (B) Representative images of Masson's trichrome and Sirius Red staining show that knockdown and overexpression of Crnde regulates collagen deposition. Scale bar = 100 μm. (C) Quantification of total fibrosis area using image‐pro plus. Four weeks after dietary manipulation to induce hyperlipidemia, echocardiography showed that the expression of Crnde interfered with LVEF (D) and FS (E). All values are expressed as mean ± SD. **P < 0.01.

To investigate the role of Crnde in cardiac fibrosis, a mouse DCM cardiac fibrosis was first established as above. Then, the Crnde‐specific short hairpin RNA (shCrnde) AAV were injected into the tail vein to knock down the Crnde expression in mice. At the same time, the AAV carrying Crnde expression element to overexpress Crnde in mice was also used. Twelve weeks after DCM models were established, qPCR was used to identify the expression of Crnde in myocardium. qPCR results displayed that the expression of Crnde in mouse myocardium was successfully increased or knocked down by respective AAV (Fig. 2A). Then, the cardiac fibrosis was detected using Masson and Sirius Red staining. The results showed that collagen deposition was significantly reduced after overexpression of Crnde (Fig. 2B,C). Conversely, after Crnde knockdown, collagen deposition was significantly elevated compared to the DCM group (Fig. 2B,C). Echocardiography showed that Crnde overexpression increased left ventricular ejection fraction (LVEF%; Fig. 2D) and shortened fraction (FS%; Fig. 2E). In contrast, Crnde knockdown attenuated LVEF and FS (Fig. 2D,E).

### Crnde negatively regulated TGF‐β‐induced myofibroblastic marker gene expression

**Figure 3:**
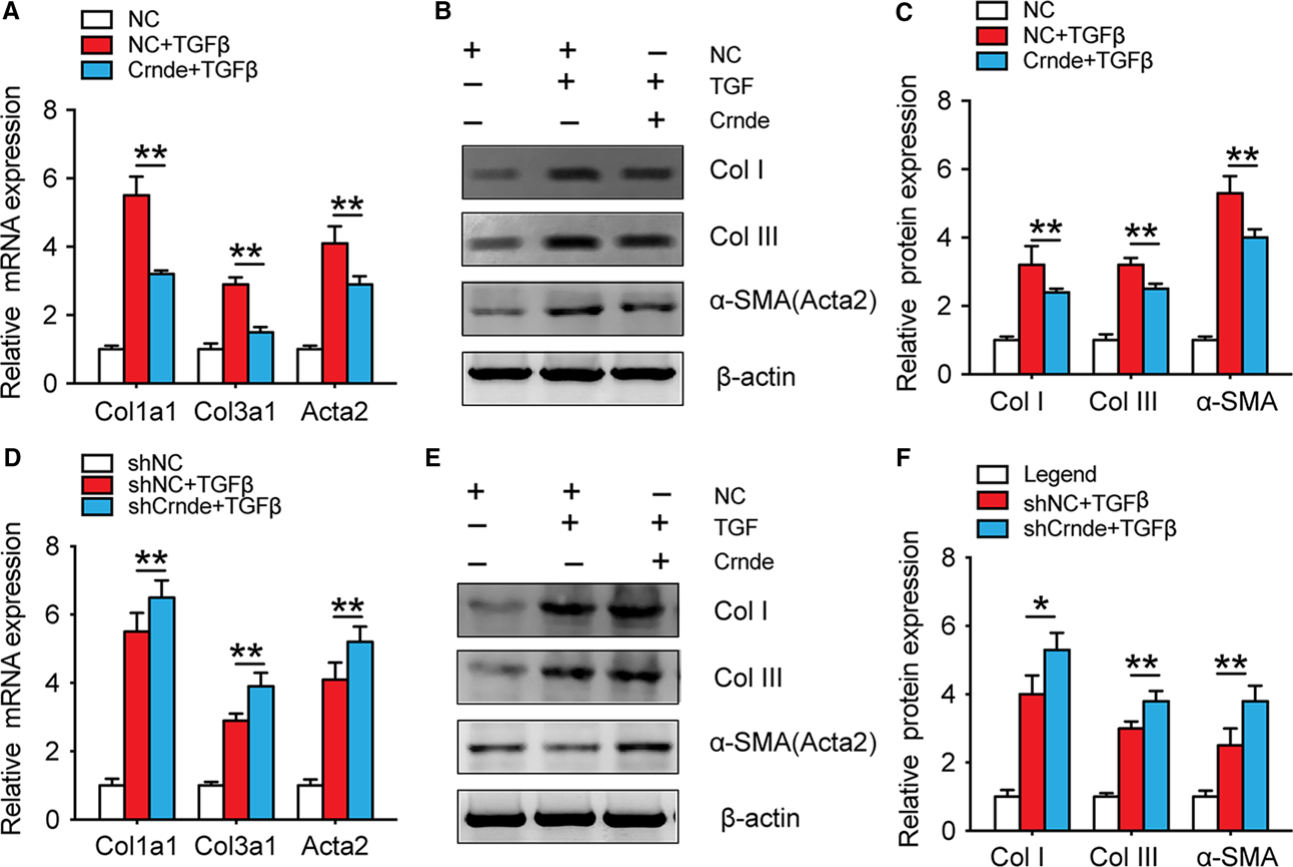
Crnde negatively regulated TGF‐β‐induced myofibroblastic marker gene expression. (A) RT‐qPCR quantification after Crnde overexpression showed a significant decrease in Col1a1, Col3a1, and Acta2 mRNA levels. (B) Western blot representative images of ColI, ColIII, and α‐SMA proteins. (C) Western blot analysis of Crnde overexpression showed a significant decrease in ColI, ColIII, and α‐SMA protein levels. (D) qPCR quantification results after Crnde knockdown showed a significant increase in Col1a1, Col3a1, and Acta2 mRNA levels. (E) Western blot representative images of ColI, ColIII, and α‐SMA proteins for Crnde knockdown. (F) Western blot analysis showed that ColI, ColIII, and α‐SMA protein levels were significantly increased after Crnde knockdown. All values are expressed as mean ± SD. *P < 0.05, **P < 0.01.

Isolated and cultured CFs were infected with AAV for overexpressing and knocking down Crnde, respectively. Then, the cells were stimulated with TGF‐β. Overexpression of Crnde significantly reduced the mRNA expression of TGFβ‐induced fibrosis marker genes Col1a1, collagen type III alpha 1 (Col3a1), and Acta2 (Fig. 3A). Similarly, protein expression results for Col1I, ColIII, and α‐SMA were consistent with mRNA results (Fig. 3B,C). After Crnde knockdown, the protein and mRNA expression of Col1a1, Col3a1, and Acta2 were significantly higher than those of the control group after TGF‐β treatment (Fig. 3D,E,F).

### Smad3 transcriptionally activated Crnde. However, Crnde inhibited the binding of Smad3 to the α‐SMA gene promoter

**Figure 4:**
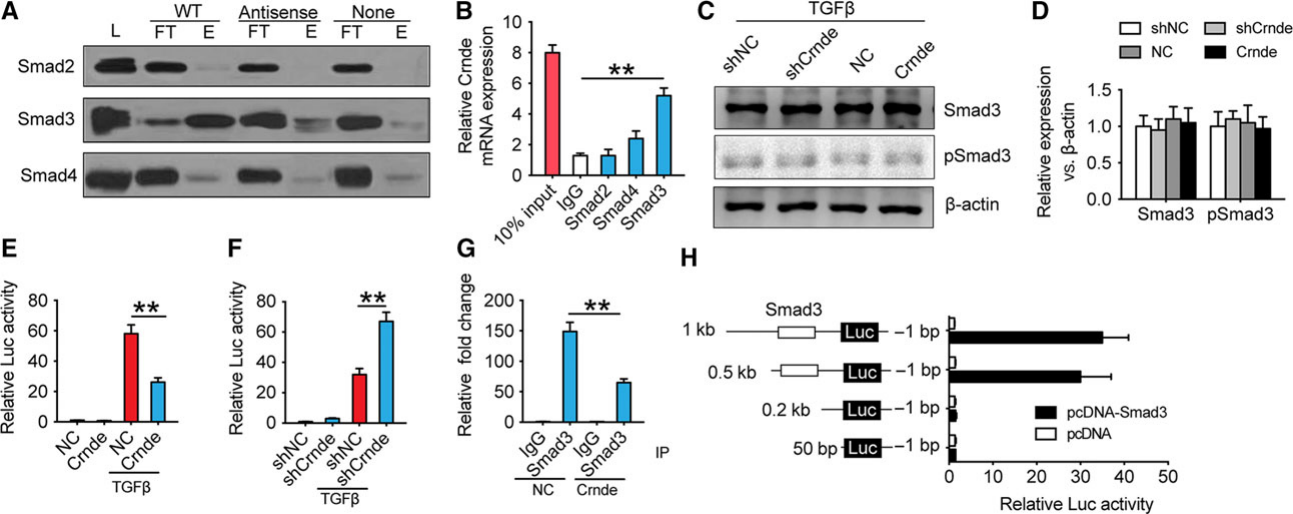
Crnde inhibits the binding of Smad3 to the α‐SMA gene promoter. (A) Representative images of Crnde and Smad2/3/4 RNA pull‐down. (B) Relative quantitative analysis of Crnde banding to Smad2/3/4 using the RIP method. (C) Representative images of Smad3 and phosphorylated Smad3 Western blot after knocking down or overexpress Crnde. (D) Quantitative analysis of (C). CFs were transfected with the α‐SMA promoter luciferase reporter gene and then infected with adenovirus NC or Crnde (E) or infected control AAV shRNA (shNC) or shCrnde (F). After TGF‐β treatment, luciferase assay was performed. (G) Crnde overexpression inhibits the binding of Smad3 to SBE in the α‐SMA promoter as measured by ChIP assay. (H) 293FT cells were cotransfected with a Crnde promoter luciferase reporter vector with or without a Smad3‐banding site and a Smad3 expression vector. Then, the luciferase assay was performed. All values are expressed as mean ± SD. **P < 0.01.

Since TGFβ‐Smad2/3/4 is the potent signaling pathway in myofibroblast differentiation of CFs, we used RNA pull‐down to detect whether Crnde interacts with Smad2, Smad3, and Smad4, directly. RNA pull‐down results showed that Crnde could bind to Smad3 (Fig. 4A). Then, RNA‐binding protein immunoprecipitation (RIP) was performed to verify the above results. RIP analysis showed that Smad3, but not Smad2/4, directly bound Crnde (Fig. 4B). Most interestingly, there was no change in the effect of Crnde on Smad3 protein and phosphorylation in TGFβ‐induced myofibroblast differentiation (Fig. 4C,D). It is known that Smad3 binds to the promoter region of Acta2 to regulate the expression of α‐SMA protein. By detection of the promoter activity of Acta2, overexpression of Crnde significantly inhibited TGF‐β‐induced α‐SMA promoter activity (Fig. 4E). Conversely, TGF‐β‐induced α‐SMA promoter activity was significantly increased after knockdown of Crnde (Fig. 4F). TGF‐β‐induced α‐SMA promoter activity is mediated by Smad3 binding to Smad‐binding element (SBE). Next, we verified whether Crnde inhibits promoter activity by inhibiting Smad3 from binding to the SBE in the promoter. ChIP analysis demonstrated that Crnde did attenuate the binding of Smad3 to SBE in the α‐SMA promoter (Fig. 4G). Interestingly, we found that there is a Smad3‐binding site at 227 bp upstream of the transcription initiation site of Crnde through the ChIPbase V2 database. By detecting the transcriptional activity of Smad3 at different positions of Crnde promotor, we demonstrated that Smad3 could transcriptionally activate Crnde (Fig. 4H).

### Crnde blocked the transcriptional activity of Smad3 via rSBEs

**Figure 5:**
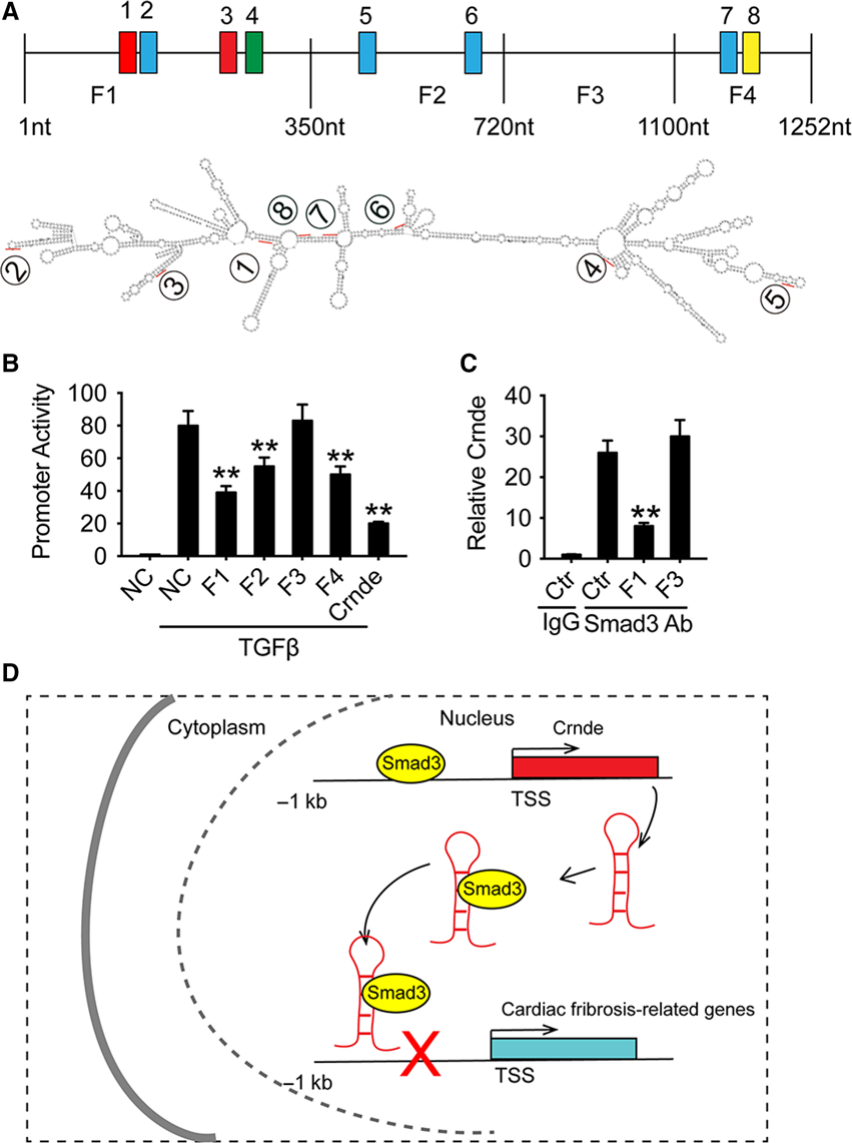
Crnde blocks the transcriptional activity of Smad3 via rSBEs. (A) Schematic location of the Crnde fragment of the mouse. The secondary structure of Crnde was predicted. The position of the digitally marked rSBEs element. (B) Effect of different Crnde fragments with or without rSBEs on α‐SMA promoter activity. (C) Quantitative analysis of Crnde bound to Smad3 by qPCR. RIP analysis showed that F1 containing rSBEs prevented binding of endogenous Crnde‐Smad3. All values are expressed as mean ± SD. (D) Schematic diagram of CRNDE inhibiting cardiac fibrosis. **P < 0.01 compared to the control group.

Since Crnde binds to Smad3 and competitively blocks the binding of Smad3 to SBE in the α‐SMA promoter, we analyzed potential binding elements in Crnde. The results showed that eight RNA SBEs (rSBEs) were observed in the Crnde RNA sequence (Fig. 5A). We used the RNAalifold Web site (http://rna.tbi.univie.ac.at/cgi-bin/RNAWebSuite/RNAalifold.cgi) to prophesy the secondary structure of Crnde and analyzed the potential location of SBE. This finding prompted us to hypothesize that Crnde can competently bind Smad3 through rSBEs, thereby preventing Smad3 binding to SBE DNA in the promoter of TGF‐β target gene. To validate this assumption, we truncated Crnde to four distinct fragments and cotransfected each fragment with the luciferase reporter gene of the α‐SMA promoter into CFs with or without rSBEs. The results showed that F1, F2, and F4 significantly inhibited TGF‐β‐induced promoter activity (Fig. 5B). To discover whether Smad3 binds specifically to rSBEs in Crnde, we transfected F1 and F3 (with or without rSBE) into CFs, respectively, and detected whether those fragments influenced the binding of Smad3 to endogenous Crnde. The results showed that F1 instead of F3 reduces the binding of Crnde to Smad3 (Fig. 5C).

## Discussion

In our current study, we identified a conserved lncRNA specifically expressed in the heart, Crnde. Moreover, in DCM, Crnde expression was significantly upregulated. Our results show that Crnde overexpression improves heart function and remodeling in DCM mice. Therefore, cardiac Crnde secretion may play an important role in the inhibition of DCM‐induced cardiac fibrosis.

Hyperglycemia‐induced ECM overproduction could alter the structure and function of the DCM heart 26. By knockdown and overexpressing Crnde, we noticed a remarkable decrease in collagen deposition. Therefore, the Smad3‐Crnde negative feedback pathway is an important mechanism of myocardial remodeling and fibrosis in DCM, and overexpression of Crnde could alleviate cardiac fibrosis in DCM.

Evidence indicated that high glucose can boost the transcription of TGF‐β gene, leading to an increase in the level of TGF‐β 3. The activated TGF‐β/SMAD signaling pathway advances the differentiation of CFs into myofibroblasts and promotes the excessive deposition of collagen fibers 27. TGF‐β is thought to produce effective cytokines/growth factors, activate fibroblasts, and promote the production of ECM in injured or diseased tissues 28, 29. TGF‐β binds to heterodimeric receptors, which are formed by TGF‐β type I and type II semi‐receptors in the plasmalemma. It co‐induces the phosphorylation of Smad2 and Smad3 transcription factors in mediating classical pathway. Phosphorylated Smad2 and Smad3 combine with Smad4 in cytoplasm and transfer together to nucleus to induce gene transcription 27. In this study, we found that the expression of Crnde was mainly enriched in CFs.

Further, *in vitro* experiments demonstrated that Crnde inhibited the differentiation of myofibroblasts from CFs and blocked transcriptional activation of Smad3. As for the mechanism, we found that Crnde interacted directly with TGF‐β/Smad3 signaling pathway and they acted as a Smad3‐Crnde feedback loop. Smad3 transcription activated Crnde. Furthermore, Crnde, in turn, inhibited Smad3 transcription of the target genes. Revealing this delicate negative feedback regulation may provide new ideas and perspectives for the treatment of DCM cardiac fibrosis.

Recent studies have shown that lncRNAs play a decisive role in promoting cardiac fibrosis. Besides, knocking down the expression of these lncRNAs could attenuate cardiac fibrosis and imporve cardiac function. Tao *et al*. indicated that H19 negatively modulated the expression of DUSP5 gene in CFs and fibrotic tissues. In freshly isolated murine CFs, upregulation of H19 expression responds to the treatment of TGF‐β. At the same time, ectopic overexpression of H19 decreased the abundance of dusp5 and increased the proliferation of CFs. Moreover, H19 silencing induced the opposite effect 30. Myocardial infarction‐associated transcripts (MIAT) can lead to myocardial infarction (MI) risk. The pathophysiological role and potential mechanism of MIAT in regulating myocardial fibrosis have been elucidated by Qu *et al*. In mouse CFs model treated with serum or Ang II, MIAT was significantly upregulated, which was accompanied by cardiac interstitial fibrosis. The upregulation of MIAT in MI is accompanied by the imbalance of some fibrosis‐related regulatory factors, such as mir‐24 (down‐regulation), furin, and TGF‐β1 (upregulation).

However, siRNA‐mediated endogenous MIAT knockout lowered myocardial fibrosis and reestablished the dysregulation of fibrosis‐related regulatory factors 31. Similarly, Wisrier expression is associated with fibrosis in cardiac mouse model and human tissue of a human patient with aortic stenosis.

The *in vitro* loss of function using modified antisense oligonucleotides indicates that wisper is a special regulator of proliferation, migration, and survival of CFs. Therefore, *in vivo* knockout of wisper can alleviate MI‐induced fibrosis and cardiac dysfunction. Functionally, wisper regulates the gene expression process of CFs, which is essential for cell characteristics, ECM deposition, proliferation, and survival 21. As mentioned above, our study mainly concentrated on the role of lncRNA in DCM‐induced cardiac fibrosis. This is exactly the opposite of the research ideas of other studies mentioned above. In this study, we found that Crnde inhibits cardiac fibrosis in DCM. Furthermore, we consider the use of Crnde for cardiac fibrosis protection strategies in DCM to overexpress Crnde in CFs.

Although tens of thousands of lncRNAs have been found in humans, many of them do not show typical high interspecific conservativeness of protein‐coding genes 32. In previous studies, mice models were usually used in many research, and researchers failed to find their homologous sequences in the human genome. However, Crnde is also present in the human genome and is also specifically expressed in the heart. Therefore, Crnde has a great value as an intervention target for DCM in the treatment of myocardial fibrosis, clinically.

In summary, this study identifies a cardiac‐specific, CFs‐enriched lncRNA. Overexpression of Crnde *in vivo* attenuates cardiac fibrosis and improves cardiac function in DCM mice. The expression of Crnde was governed by Smad3. However, Crnde also inhibited the transcriptional activation of Smad3 on target genes, thereby inhibiting the differentiation of myofibroblasts from CFs during DCM‐induced cardiac fibrosis. All in all, our findings offer some new views into the clinical development of anti‐cardiac fibrosis strategies.

## Materials and methods

### Mouse neonatal cardiac fibroblasts (CFs) isolation and culture

The hearts of C57BL/6 mice, 1–3 days old, were minced and put into 0.25% trypsin. Mixed cell suspensions were centrifuged and resuspended in Dulbecco modified Eagle's medium supplemented with 10% FBS, 100 U·mL^−1^ penicillin, and 100 μg·mL^−1^ streptomycin.

The suspension was inoculated on the flask for 90 min, and fibroblasts attached to the bottom of the flask preferentially. Nonadherent and weakly adherent cells were thought to be CM and transferred to new culture flasks for subsequent research. CFs grew to the confluence and was subcultured with trypsin. CFs and CM were incubated at 37 °C/5% CO_2_. The CFs (second and third generation) were studied. After starving for 24 h in serum‐free medium, recombinant human TGF‐β1 (10 ng·mL^−1^, Sigma, St. Louis, MO, USA) was administered to CFs for 24 h.

### Animals

Male C57BL/6 mice, weighing 25–30 g, were housed in a cage for every five (Sichuan University Animal Center, Chengdu, China). The mice were randomly given a high‐fat diet for 4 weeks. After 4 weeks of hyperlipidemia induced by dietary manipulation, mice were injected intraperitoneally with low dose of streptozotocin (STZ, 35 mg·kg^−1^ body weight), dissolved in 0.1 mmol·L^−1^ citrate phosphate buffer (pH 4.5), and immediately given to avoid degradation. A week later, the fasting blood glucose (FBG) was measured with a portable blood glucose meter (One Touch Sure Step Meter; Life Scan Co., Milpitas, CA, USA). Mice with FBG levels above 16.7 mmol·L^−1^ were considered diabetic and were selected for further study. One week after the injection of STZ, mice were injected with AAV or NC. Animals were randomized into six groups of eight mice each: sham operation (sham), DCM, DCM infected with negative control AAV for Crnde knockdown (DCM + shNC), DCM infected with AAV for Crnde knockdown (DCM + shCrnde), DCM infected with negative control AAV for Crnde overexpression (DCM + NC), DCM infected with AAV for Crnde overexpression (DCM + Crnde). Mice were sacrificed 12 weeks after STZ injection. All procedures were approved by the ethical‐scientific committee of The General Hospital of Western Theater Commad, which strictly conforms to the Guide for the Care and Use of Laboratory Animals.

### Cardiac ultrasound

Mice were anesthetized with isoflurane and taken in the supine position. The VEVO 770 high‐resolution system with probe at the frequency of 40‐MHz RMV 704 was used for echocardiographic examination to record left ventricular end‐systolic diameters (LVESd) and left ventricular end‐diastolic (LVEDd) values. LVEF and left ventricular fractional shorting (LVFS) are automatically calculated by an echocardiographic instrument. The calculation formula is: LVFS (%) = (LVEDd − LVESd)/LVEDd × 100, EF (%) = (LVEDd3 − LVESd3)/LVEDd3 × 100.

### Real‐time quantitative PCR

The RNAiso Plus reagent was used for RNA extraction (Takara, Kusatsu, Shiga, Japan). cDNA was prepared from 1 μg of total RNA using PrimeScriptTM RT reagent Kit with gDNA Eraser (Takara). The PCR amplifications were carried out using specific primers for each gene as follows: Col1a1 (F) 5′‐GCTCCTCTTAGGGGCCACT‐3′, (R) 5′‐CCACGTCTCACCATTGGGG‐3′; Col3a1 (F) 5′‐CTGTAACATGGAAACTGGGGAAA‐3′, (R) 5′‐CCATAGCTGAACTGAAAACCACC‐3′; fibronectin 1 (F) 5′‐ATGTGGACCCCTCCTGATAGT‐3′, (R) 5′‐GCCCAGTGATTTCAGCAAAGG‐3′; Acta2 (F) 5′‐GTCCCAGACATCAGGGAGTAA‐3′, (R) 5′‐TCGGATACTTCAGCGTCAGGA‐3′; Crnde (F) 5′‐CTCTGGTCCAGGACGAAGACT‐3′, (R) 5′‐CCTTTGCATCTCGATGGGAAC‐3′.

### Western blotting analysis

CFs and heart samples were lysed using RIPA buffer (Beyotime, Shanghai, China) containing a protease inhibitor cocktail (Sigma). The concentration of the protein sample was assessed by Bicinchoninic Acid Protein Assay Kit (Thermo Fisher, Carlsbad, CA, USA). The same amount of protein was separated from SDS/PAGE and blotted onto poly(vinylidene difluoride) membrane. The main antibodies were from the following sources: α‐SMA (1 : 1000; Sigma), p‐Smad3 (1 : 1000; CST), Smad3 (1 : 1000; CST), Col1a1 (1 : 1000; CST, Waltham, MA, USA), Col3a1 (1 : 1000; CST), Smad2 (1 : 1000; CST), Smad4 (1 : 1000; CST), and β‐actin (1 : 1000; Bioss, wuhan, China). ECL chemiluminescence kit (Thermo Fisher) was used to observe all proteins. Bio‐Rad, Hercules, CA, USA software was used to quantify each band, and β‐actin was used as load control.

### AAV construct

A self‐complementary recombinant AAV (subtype 9) was constructed using AAV‐Helper‐Free System (Miaoling Technologies, Wuhan, China). For expression of Crnde, the full length of Crnde was inserted into the AAV‐GFP plasmid (Biotek Technologies, Shanghai, China). For knockdown of Crnde, the specific short hairpin RNA (shRNA) was inserted into the AAV‐U6‐shRNA‐RFP plasmid (Biotek Technologies). Recombinant AAV containing Crnde or shRNA were generated via cotransfection of AAV‐Crnde‐GFP (AAV‐U6‐shCrnde‐RFP), pHelper, and pAAV‐RC5 into 293FT cells using polyethyleneimine. The AAV‐GFP and AAV‐U6‐shRNA‐RFP were constructed as a control virus. After 72 h of transfection, virus particles were isolated, purified, and quantitatively analyzed. Three days after operation, mice were injected with AAV‐shCrnde (or AAV‐shNC) and AAV‐Crnde (or AAV‐NC) via tail vein (1 × 10^10^/μL, 200 μL). Mice in sham‐operated group and MI group underwent the same procedure, but received 200 mL saline.

### Assessment of cardiac fibrosis

Hearts were collected from sham and DCM mice to eliminate atria and large vessels. Heart tissue was fixed overnight with 4% formalin. Paraffin tissue sections were processed according to standard histological procedures and used for Mason trichrome staining and Sirius red staining. Fibrotic tissue was quantified by image‐pro plus 6.0 (Media Cybernetics, Inc. Rockville, MD, USA). Three slices were used for each animal. Each slide analyses five regions, each divided into 100 squares. Collagen staining (blue staining) scored 1 (presence) or 0 (absence) in each square. The results showed that the area of fibrosis accounted for a percentage of the total area.

### RNA pull‐down

Mouse Crnde full‐length RNA and Crnde antisense RNA were synthesized and labeled with biotin‐UTP by an *in vitro* transcription kit (Roche, Boston, MA, USA). For the Smad3 pull‐down assay, 1 μg of biotinylated Crnde RNA or Crnde antisense RNA was transfected into CFs in a 10 cm dish and incubated overnight at 37 °C.

By adding 1% formaldehyde to cross‐linked cells, the cells were dissolved for 30 min at 4 °C in FA solution buffer containing 40 U·mL^−1^ RNase inhibitor (Sigma) and 1× Haltm protease inhibitor mixture (Thermo). Cell lysates were treated with DNase I at 37 °C for 20 min, and then rotated at maximum speed for 10 min. Then, the supernatant (about 200 mL) and 20 μL avidin‐coated agarose beads (Thermo) were cultured at 25 °C for 1 h.

After four washes with washing/binding buffer (PBS containing 0.1% SDS and 1% Nonidet P‐40 or 0.5% sodium deoxycholate), the bead‐RNA‐protein complex was spin‐precipitated, and Smad3 was detected by western blotting. In the smad3‐gas5 binding experiment *in vitro*, the labeled GAS5 F1 fragment (30 ng) and 0.8 UG recombinant Smad3 (Sigma‐Aldrich) were incubated overnight at 4 °C in 50 μL PBS. The next day, the RNA protein complex was pulled down by 50 μL avidin‐coated agarose beads. Smad2, Smad3, and Smad4 were detected by western blotting after washing with washing/binding buffer (PBS containing 0.1% SDS and 1% Nonidet P‐40 or 0.5% sodium deoxycholate) for three times.

### RIP assay

RIP assay is performed as described above. Fixed 80–90% of the fusion cells on a 15 cm culture dish with 1% polyformaldehyde, then scraped centrifugation in FA containing 40 units·mL^−1^ RNase inhibitor and 1× HaltTM protease inhibitor cocktail. Lysis in the liquid. After 4–6 rounds of 50% power output ultrasound treatment, 300 μL whole‐cell lysate was incubated overnight with IgG, Smad2, Smad3, or Smad4 antibodies (1 μg) of normal rabbits at 4 °C. The immunoprecipitation was captured by protein A/G‐agarose beads (50 μL) the next day. After washing with FA solution buffer, protease K was cultured at 42 °C for 1 h to digest protein. Then, the RNA of immunoprecipitation was separated. The purified RNA was analyzed by qRT‐PCR.

### ChIP assay

The cells were fixed with 1% formaldehyde. The cross‐linked cells were collected by centrifugation at 4 °C and washed with PBS containing a protease inhibitor before final collection. Then, the cells were rotated in 4% SDS lysis buffer for 20 min and resuspended at 4 °C. Then, the DNA was cut into 500–1000 bp fragments by sonication on ice. The lysate was immunoprecipitated by co‐immunoprecipitation reagent (Milipore, St. Louis, MO, USA) with IgG (NC) or Smad3 antibody of 2 μg. The promoter region of a‐SMA gene containing SBE was amplified by qPCR.

### Luciferase reporter gene assay

Two hundred and fifty nanograms of empty vector or pcDNA 3.0‐crnde and 250 ng fluorescein reporter vector driven by a‐SMA promoter (pgl3‐basic; Promega, San Luis Obispo, CA, USA) were cotransfected into CFS with Lipofectamine 3000 (Invitrogen, Carlsbad, CA, USA) in 12 orifice plates. Cells were treated with 5 ng·mL^−1^ TGF‐β1 for 8 h. According to the manufacturer's protocol, luciferase activity was measured by using a Luciferase Assay Kit (Promega). The experiment was repeated three times in triplicate. The transcriptional activation of Smad3 on Crnde was examined. Liposome 3000 (Invitrogen) was used to cotransfect 250 ng empty vector or pcDNA 3.0‐smad3 and 250 ng luciferase reporter vector (driven by different stages of Crnde promoter) into 12‐well plates CFs. The experiment was repeated three times in triplicate.

### Statistical analysis

All values are expressed as mean ± SD. The comparison of the parameters between the two groups was performed by an unpaired Student's *t*‐test. One‐way ANOVA followed by Student–Newman–Keuls post hoc test was used to determine the significance of differences between results. *P* values ranging from 0.01 to 0.05 and below 0.01 are considered significant (*) and very significant (**), respectively. The sample size or experiment size is determined according to experience to obtain sufficient statistical power. No data points were excluded from all experiments in this study. Randomization was not used in this study.

## Conflict of interest

The authors declare no conflict of interest.

## Author contributions

JH, DZ, and JL designed this study; YZ, YH, LX, and WX finished the animal studies; DZ and JL finished the CFs and CMs isolations; JH, QZ, and CR analyzed data; JH, DZ, and YZ performed western blot, qPCR, and staining.

## References

[1] Sun B , He F , Sun L , Zhou J , Shen J , Xu J , Wu B , Liu R , Wang X , Xu H *et al* (2019) Cause‐specific risk of major adverse cardiovascular outcomes and hypoglycemic in patients with type 2 diabetes: a multicenter prospective cohort study. Endocrine 63, 44–51. 30121774 10.1007/s12020-018-1715-0

[2] Kim MK , Jeong JS , Yun JS , Kwon HS , Baek KH , Song KH , Ahn YB & Ko SH (2018) Hemoglobin glycation index predicts cardiovascular disease in people with type 2 diabetes mellitus: A 10‐year longitudinal cohort study. J Diabetes Complications 32, 906–910. 30121206 10.1016/j.jdiacomp.2018.08.007

[3] Yue Y , Meng K , Pu Y & Zhang X (2017) Transforming growth factor beta (TGF‐beta) mediates cardiac fibrosis and induces diabetic cardiomyopathy. Diabetes Res Clin Pract 133, 124–130. 28934669 10.1016/j.diabres.2017.08.018

[4] Li B , Zheng Z , Wei Y , Wang M , Peng J , Kang T , Huang X , Xiao J , Li Y & Li Z (2011) Therapeutic effects of neuregulin‐1 in diabetic cardiomyopathy rats. Cardiovasc Diabetol 10, 69. 21798071 10.1186/1475-2840-10-69 PMC3170868

[5] Travers JG , Kamal FA , Robbins J , Yutzey KE & Blaxall BC (2016) Cardiac fibrosis: the fibroblast awakens. Circ Res 118, 1021–1040. 26987915 10.1161/CIRCRESAHA.115.306565 PMC4800485

[6] Farris SD , Don C , Helterline D , Costa C , Plummer T , Steffes S , Mahr C , Mokadam NA & Stempien‐Otero A (2017) Cell‐specific pathways supporting persistent fibrosis in heart failure. J Am Coll Cardiol 70, 344–354. 28705316 10.1016/j.jacc.2017.05.040

[7] Dong S , Ma W , Hao B , Hu F , Yan L , Yan X , Wang Y , Chen Z & Wang Z (2014) microRNA‐21 promotes cardiac fibrosis and development of heart failure with preserved left ventricular ejection fraction by up‐regulating Bcl‐2. Int J Clin Exp Pathol 7, 565–574. 24551276 PMC3925900

[8] Xiang FL , Fang M & Yutzey KE (2017) Loss of beta‐catenin in resident cardiac fibroblasts attenuates fibrosis induced by pressure overload in mice. Nat Commun 8, 712. 28959037 10.1038/s41467-017-00840-w PMC5620049

[9] Zhou Y , Richards AM & Wang P (2017) Characterization and standardization of cultured cardiac fibroblasts for *ex vivo* models of heart fibrosis and heart ischemia. Tissue Eng Part C Methods 23, 422–433. 28514938 10.1089/ten.TEC.2017.0169

[10] Cavin S , Maric D & Diviani D (2014) A‐kinase anchoring protein‐Lbc promotes pro‐fibrotic signaling in cardiac fibroblasts. Biochim Biophys Acta 1843, 335–345. 24269843 10.1016/j.bbamcr.2013.11.008

[11] Fujikawa T , Fujita R , Iwaki Y , Matsumura S , Fushiki T & Inoue K (2010) Inhibition of fatty acid oxidation activates transforming growth factor‐beta in cerebrospinal fluid and decreases spontaneous motor activity. Physiol Behav 101, 370–375. 20619281 10.1016/j.physbeh.2010.06.006

[12] Shih YC , Chen CL , Zhang Y , Mellor RL , Kanter EM , Fang Y , Wang HC , Hung CT , Nong JY , Chen HJ *et al* (2018) Endoplasmic reticulum protein TXNDC5 augments myocardial fibrosis by facilitating extracellular matrix protein folding and redox‐sensitive cardiac fibroblast activation. Circ Res 122, 1052–1068. 29535165 10.1161/CIRCRESAHA.117.312130 PMC5899016

[13] He X , Gao X , Peng L , Wang S , Zhu Y , Ma H , Lin J & Duan DD (2011) Atrial fibrillation induces myocardial fibrosis through angiotensin II type 1 receptor‐specific Arkadia‐mediated downregulation of Smad7. Circ Res 108, 164–175. 21127293 10.1161/CIRCRESAHA.110.234369 PMC3035429

[14] Khalil H , Kanisicak O , Prasad V , Correll RN , Fu X , Schips T , Vagnozzi RJ , Liu R , Huynh T , Lee SJ *et al* (2017) Fibroblast‐specific TGF‐beta‐Smad2/3 signaling underlies cardiac fibrosis. J Clin Invest 127, 3770–3783. 28891814 10.1172/JCI94753 PMC5617658

[15] Kunamalla A , Ng J , Parini V , Yoo S , McGee KA , Tomson TT , Gordon D , Thorp EB , Lomasney J , Zhang Q *et al* (2016) Constitutive expression of a dominant‐negative TGF‐beta type II receptor in the posterior left atrium leads to beneficial remodeling of atrial fibrillation substrate. Circ Res 119, 69–82. 27217399 10.1161/CIRCRESAHA.115.307878 PMC4920729

[16] Sibinska Z , Tian X , Korfei M , Kojonazarov B , Kolb JS , Klepetko W , Kosanovic D , Wygrecka M , Ghofrani HA , Weissmann N *et al* (2017) Amplified canonical transforming growth factor‐beta signalling via heat shock protein 90 in pulmonary fibrosis. Eur Respir J 49, 1501941. 28182573 10.1183/13993003.01941-2015

[17] Long J , Badal SS , Ye Z , Wang Y , Ayanga BA , Galvan DL , Green NH , Chang BH , Overbeek PA & Danesh FR (2016) Long noncoding RNA Tug1 regulates mitochondrial bioenergetics in diabetic nephropathy. J Clin Invest 126, 4205–4218. 27760051 10.1172/JCI87927 PMC5096930

[18] Morchikh M , Cribier A , Raffel R , Amraoui S , Cau J , Severac D , Dubois E , Schwartz O , Bennasser Y & Benkirane M (2017) HEXIM1 and NEAT1 long non‐coding RNA form a multi‐subunit complex that regulates DNA‐mediated innate immune response. Mol Cell 67, 387–399.e5. 28712728 10.1016/j.molcel.2017.06.020

[19] St Laurent G , Wahlestedt C & Kapranov P (2015) The landscape of long noncoding RNA classification. Trends Genet 31, 239–251. 25869999 10.1016/j.tig.2015.03.007 PMC4417002

[20] Creemers EE & van Rooij E (2016) Function and therapeutic potential of noncoding RNAs in cardiac fibrosis. Circ Res 118, 108–118. 26538569 10.1161/CIRCRESAHA.115.305242

[21] Micheletti R , Plaisance I , Abraham BJ , Sarre A , Ting CC , Alexanian M , Maric D , Maison D , Nemir M , Young RA *et al* (2017) The long noncoding RNA Wisper controls cardiac fibrosis and remodeling. Sci Transl Med 9, eaai9118. 28637928 10.1126/scitranslmed.aai9118 PMC5643582

[22] Piccoli MT , Gupta SK , Viereck J , Foinquinos A , Samolovac S , Kramer FL , Garg A , Remke J , Zimmer K , Batkai S *et al* (2017) Inhibition of the cardiac fibroblast‐enriched lncRNA Meg3 prevents cardiac fibrosis and diastolic dysfunction. Circ Res 121, 575–583. 28630135 10.1161/CIRCRESAHA.117.310624

[23] Zhang Y , Zhang YY , Li TT , Wang J , Jiang Y , Zhao Y , Jin XX , Xue GL , Yang Y , Zhang XF *et al* (2018) Ablation of interleukin‐17 alleviated cardiac interstitial fibrosis and improved cardiac function via inhibiting long non‐coding RNA‐AK081284 in diabetic mice. J Mol Cell Cardiol 115, 64–72. 29305939 10.1016/j.yjmcc.2018.01.001

[24] Zhou KR , Liu S , Sun WJ , Zheng LL , Zhou H , Yang JH & Qu LH (2017) ChIPBase v2.0: decoding transcriptional regulatory networks of non‐coding RNAs and protein‐coding genes from ChIP‐seq data. Nucleic Acids Res 45, D43–D50. 27924033 10.1093/nar/gkw965 PMC5210649

[25] Hou M , Tian F , Jiang S , Kong L , Yang D & Gao G (2016) LocExpress: a web server for efficiently estimating expression of novel transcripts. BMC Genom 17, 1023. 10.1186/s12864-016-3329-3 PMC5260097 28155723

[26] Wang WK , Wang B , Lu QH , Zhang W , Qin WD , Liu XJ , Liu XQ , An FS , Zhang Y & Zhang MX (2014) Inhibition of high‐mobility group box 1 improves myocardial fibrosis and dysfunction in diabetic cardiomyopathy. Int J Cardiol 172, 202–212. 24485636 10.1016/j.ijcard.2014.01.011

[27] Blyszczuk P , Muller‐Edenborn B , Valenta T , Osto E , Stellato M , Behnke S , Glatz K , Basler K , Luscher TF , Distler O *et al* (2017) Transforming growth factor‐beta‐dependent Wnt secretion controls myofibroblast formation and myocardial fibrosis progression in experimental autoimmune myocarditis. Eur Heart J 38, 1413–1425. 27099262 10.1093/eurheartj/ehw116

[28] Araki S , Izumiya Y , Rokutanda T , Ianni A , Hanatani S , Kimura Y , Onoue Y , Senokuchi T , Yoshizawa T , Yasuda O *et al* (2015) Sirt7 contributes to myocardial tissue repair by maintaining transforming growth factor‐beta signaling pathway. Circulation 132, 1081–1093. 26202810 10.1161/CIRCULATIONAHA.114.014821

[29] Tomcik M , Palumbo‐Zerr K , Zerr P , Sumova B , Avouac J , Dees C , Distler A , Becvar R , Distler O , Schett G *et al* (2016) Tribbles homologue 3 stimulates canonical TGF‐beta signalling to regulate fibroblast activation and tissue fibrosis. Ann Rheum Dis 75, 609–616. 25603829 10.1136/annrheumdis-2014-206234

[30] Tao H , Cao W , Yang JJ , Shi KH , Zhou X , Liu LP & Li J (2016) Long noncoding RNA H19 controls DUSP5/ERK1/2 axis in cardiac fibroblast proliferation and fibrosis. Cardiovasc Pathol 25, 381–389. 27318893 10.1016/j.carpath.2016.05.005

[31] Qu X , Du Y , Shu Y , Gao M , Sun F , Luo S , Yang T , Zhan L , Yuan Y , Chu W *et al* (2017) MIAT is a pro‐fibrotic long non‐coding RNA governing cardiac fibrosis in post‐infarct myocardium. Sci Rep 7, 42657. 28198439 10.1038/srep42657 PMC5309829

[32] Johnsson P , Lipovich L , Grander D & Morris KV (2014) Evolutionary conservation of long non‐coding RNAs; sequence, structure, function. Biochim Biophys Acta 1840, 1063–1071. 24184936 10.1016/j.bbagen.2013.10.035 PMC3909678

